# Interventions to Facilitate Access to Long-Term Care for Community-Dwelling Older Adults: A Scoping Review

**DOI:** 10.1177/01640275251380534

**Published:** 2025-09-17

**Authors:** Jinbao Zhang, Alan Dargan, Wenjing Zhang, Julien Forder

**Affiliations:** 1 Personal Social Services Research Unit, 2240 University of Kent, England, UK; 2 NIHR Health and Social Care Workforce Research Unit, The Policy Institute, 4616 King’s College London, England, UK

**Keywords:** system navigation, social care, service access, early identification, integrated care

## Abstract

Accessing long-term care can be challenging due to complex care systems. This study aimed to identify evidence on interventions designed to improve access to long-term care for community-dwelling older adults. A systematic search of five databases identified 16 studies published between 2013 and 2023. Interventions were categorized into four types: early identification (n = 6), integrated care (n = 5), partnership (n = 2), and other (n = 3). Common strategies included providing information, making referrals, and providing coordinated care. Most interventions were delivered by healthcare professionals, targeted high-risk older adults, and focused on nursing home admissions, with less attention to other long-term care services. Interventions could target older adults with advanced age or specific health conditions and identify unmet needs at early stages. Future research could document intervention length, explore its impact on service access, and evaluate the feasibility and effectiveness of involving non-healthcare professionals in delivering interventions.

## Introduction

By 2050, one in six people worldwide will be aged 65 and over, leading to a growing demand for long-term care services and support (de Meijer et al., 2013; United Nations, 2019). Long-term care refers to personal care and related services for people with functional impairments, particularly those experiencing limitations in activities of daily living, such as eating, dressing, and personal hygiene (Colombo et al., 2011). Unlike post-acute care, which provides short-term support (typically lasting weeks) for recovery or transition following hospitalization, long-term care often addresses persistent needs over an extended period. In England, local authorities received an average of 3,910 care requests per day from new older clients during 2023-24 (NHS Digital, 2023). Of these requests, nearly 73% came from the community, while 26% originated from hospital discharges and other routes, such as diversion from hospital services and self-funders with depleted funds. Given the substantial volume of care requests from the community, it is essential to ensure that older adults obtain adequate and timely services and support to minimize unmet needs, eventually enhancing their well-being and reducing high-cost healthcare utilization (Huang et al., 2024).

Older adults, particularly those with functional impairments, often find it challenging to access long-term care due to the complexity of care systems. In many countries, long-term care systems are characterized by complexity, including lacking transparency and clarity in eligibility criteria and application processes (Comas-Herrera et al., 2010; Quigley et al., 2022; Xie et al., 2024). For older people, particularly those with physical or cognitive impairments, limited communication skills and reduced cognitive capacity hinder their ability to process information about eligibility criteria, service options, and fees (Baxter et al., 2021; Erlandsson et al., 2022). Without support to address these barriers, older people are often unable to obtain services they need, resulting in escalated unmet needs, greater risk for hospitalization and emergency department visits, and reduced well-being (Huang et al., 2024; Xie et al., 2024).

A growing body of research has reviewed interventions that facilitated service access. For example, Røsvik et al. (2020) conducted a scoping review and identified five categories of interventions—case management, financial support, referral enhancing, information provision, and inpatient focused programs—designed to enhance access to community care for home-dwelling individuals with dementia. Similarly, Kokorelias et al. (2023) reviewed navigation programs for people with dementia, highlighting their role in delaying institutionalization and enhancing caregiver satisfaction. However, these studies have largely focused on the use of specific subtypes of long-term care services, such as dementia-specific community services, thereby overlooking interventions to facilitate access to other long-term care (e.g., residential care). Additionally, the characteristics and mechanisms of interventions that promote access to long-term care for older adults remain unknown. Addressing these gaps is crucial to inform policymakers and practitioners in developing tailored programs, promoting service access, and ultimately improving the well-being of older adults.

The objective of this study is to identify and map existing evidence on interventions that facilitate access to long-term care services for older people living in the community. This review was guided by two research questions: (1) what are the characteristics of the interventions? And (2) what outcomes have been reported from these interventions?

To classify the components of interventions, we draw on Levesque et al.’s (2013) conceptual framework for access to healthcare. This model identifies five dimensions of access: approachability, availability and accommodation, appropriateness, acceptability, and affordability. Approachability refers to individuals’ ability to recognize that services exist and are accessible. This included components such as screening and information provision. Availability and accommodation concern whether services could be accessed in a timely and convenient manner. Relevant strategies include service referrals and practical support. Appropriateness reflects the fit between services and the specific, often complex, needs of older adults. This dimension is reflected in components such as care planning and care coordination. Acceptability pertains to individuals’ perception of services as culturally, socially, and personally suitable. Enhancing acceptability involves communication to foster trust and address concerns, as well as emotional support to encourage individuals to obtain services. Finally, affordability represents one’s economic resources (e.g., income) to use appropriate services. This dimension was not included in our scoping review because the included studies did not address it.

## Method

This review was conducted in accordance with the Joanna Briggs Institute (JBI) methodological guidance for scoping reviews (Peters et al., 2020). Before starting the scoping review, we performed a preliminary search of Medline, Prospero, the Cochrane Database of Systematic Reviews, and the JBI Evidence Synthesis. This search revealed that there were no current or ongoing scoping reviews or systematic reviews on the topic. A protocol was developed and registered in the Open Science Framework Registries (registration link: https://doi.org/10.17605/OSF.IO/PQ4UT). The Preferred Reporting Items for Systematic Reviews and Meta-Analyses extension for Scoping Reviews (PRISMA-ScR) was used for reporting (see Supplemental Material Appendix A; Tricco et al., 2018).

### Eligibility Criteria

Following JBI’s recommendations, we used the Participants, Concept, and Context framework to determine eligibility criteria. A summary of the inclusion and exclusion for this review is presented in Table 1. Participants were defined as older people aged 65 and over. Studies focusing on older people as the target group were included. The main concept in this review was interventions that facilitated access to long-term care. Long-term care typically includes home- and community-based services, residential care, and nursing home services. However, post-discharge services, such as reablement care, provided short-term (typically lasting weeks) were not classified as long-term care. Interventions were defined as techniques, strategies, or activities that aimed at promoting access to any type of long-term care services. Studies were included if the purpose or outcome of the intervention was to promote access to long-term care. This review focused on interventions implemented in home- and community-based settings, regardless of their geographic locations.

**Table 1.** Inclusion and Exclusion Criteria

Framework	Inclusion criteria	Exclusion criteria
Participants	Older people aged 65 or above as the target group	Younger people aged below 65
Concept	The purpose or outcome of interventions was facilitating access to long-term care services	1) No interventions 2) The objective or outcome of the intervention was not related to access to long-term care
Context	Home- and community-based settings	Not at home or in the community, e.g., hospitals and nursing homes
Types of evidence sources	All study designs, including quantitative, qualitative, and mixed methods	Commentaries, editorials, protocols, and conference proceedings

This review included studies with all types of study designs, including quantitative, qualitative, and mixed methods. Literature reviews (e.g., meta-analyses and systematic reviews) were included to avoid duplication of data and provide conceptual frameworks for categorizing specific interventions into some types (Peters et al., 2022). Commentaries, editorials, protocols, and conference proceedings were excluded from screening and analysis.

### Search Strategy and Study Screening

To develop a comprehensive search, we performed an initial search in Medline to test our search terms. The results were reported in our priori protocol. Based on the initial searches, we finalized the key search terms, and conducted searches across five databases: Medline, PsycINFO, CINAHL, Social Sciences Citation Index (SSCI), and the Cochrane Database of Systematic Reviews. Only studies published in English between 1^st^ January 2013 and 31^st^ July 2023 were included, as we focus on the latest evidence relevant to current policy practices. We also applied filters to specify the age of participants (65 and older) in Medline, PsycINFO, and CINAHL. The full search strategy for each database is provided in Supplemental Material Appendix B.

Following the search, all records were collated and imported into Mendeley Desktop Version 1.19.8 (Mendeley Ltd., Elsevier, Netherlands), and duplicates were removed. The final list of identified studies was then uploaded to Rayyan (Qatar Computing Research Institute, Doha, Qatar), a free web-based systematic review application that facilitates collaborative study screening. First, one reviewer (JZ) screened all records by title and excluded protocol studies. Second, the titles and abstracts of all records were independently screened by two reviewers (AD and JZ). Finally, the two reviewers independently screened full-text records and documented their reasons for inclusion and exclusion. Any discrepancies were addressed by discussions.

### Data Extraction and Synthesis

In line with the recommendations of Pollock et al. (2023) for data extraction and analysis, the research team developed a data extraction form, which was piloted and refined based on six included studies. Two researchers (AD and JZ) independently reviewed each article and extracted the following information: (1) authors, publication year, study location, and study aims; (2) methodology and participants; (3) characteristics of the intervention, including components and frequency; and (4) main findings. Any discrepancies were resolved through discussion.

A qualitative content analysis was conducted on the included articles using an inductive approach, as there was limited existing evidence or established frameworks on this topic. Initially, three team members (AD, WZ, and JZ) performed open coding on four included articles, generating an initial list of codes and possible categories of the interventions. These codes were discussed and refined to create a shared codebook. Using this codebook, the three researchers collaboratively coded the remaining articles, noting any new codes that emerged during the analysis. Finally, the first author conducted a preliminary categorization of the interventions based on the aims, components, and mechanisms of these interventions. These were classified into four types: (1) early identification that detects high-risk older adults at an early stage, (2) integrated care that coordinates healthcare and long-term care services, (3) partnership that highlights joint working among stakeholders, and (4) other types. This initial categorization was subsequently reviewed and refined by the research team to ensure consensus.

## Results

### Study Selection and Characteristics

**Figure 1.**
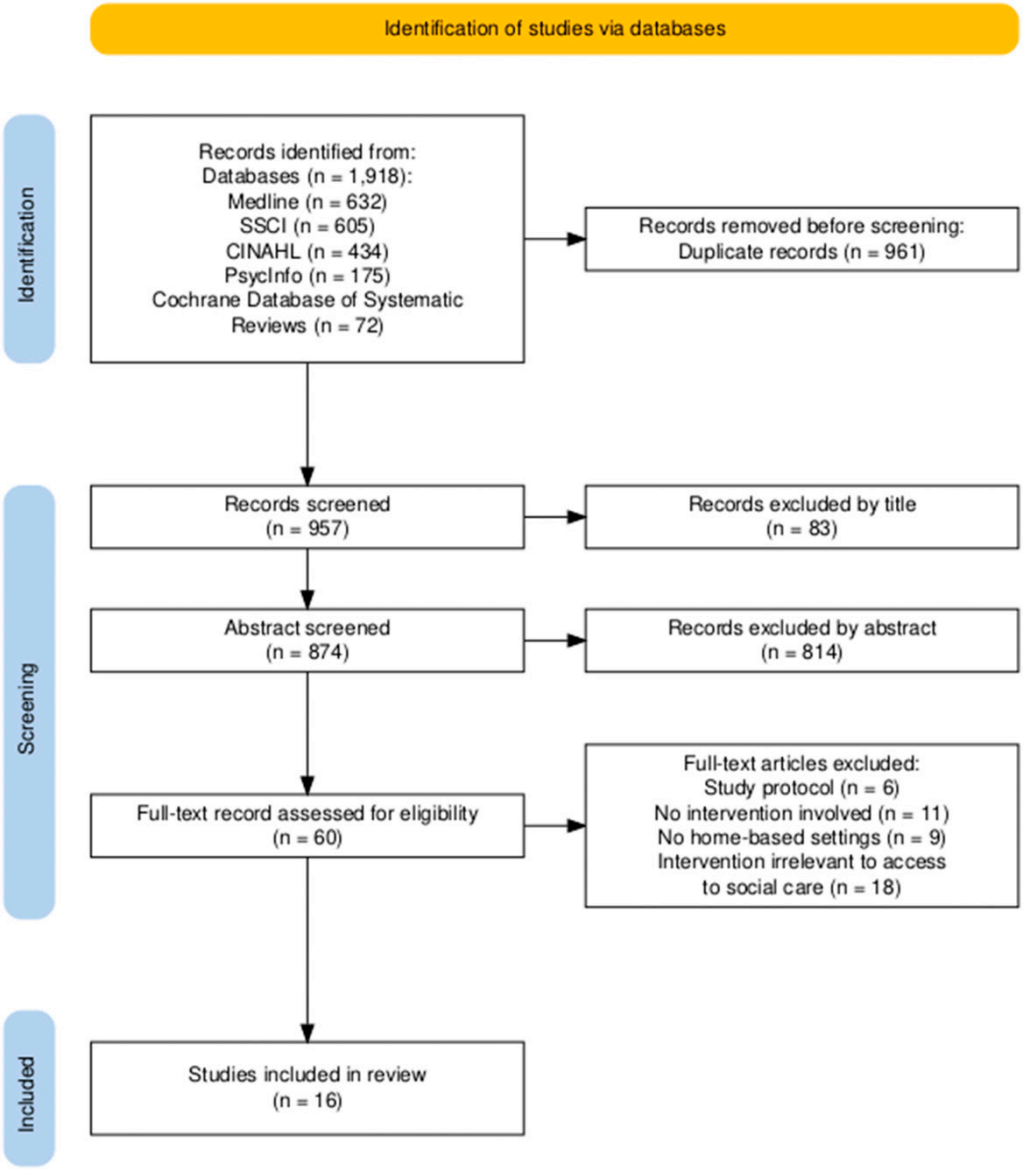
The Preferred Reporting Items for Systematic Reviews and Meta-Analyses Flow Diagram for Article Selection

A total of 1,918 studies were identified through the search, with 961 duplicate records removed. After screening by title and abstract, 897 records were excluded, leaving 60 articles for full-text screening. Finally, 16 studies were included in the data extraction process after full-text screening. The article selection process is reported in Figure 1.

Table 2 presents the characteristics of the 16 included studies, including their aims, participants, and key components of the intervention. These studies were published between 2013 and 2022, with approximately 60% published since 2019, indicating growing interest in this area. Geographically, the studies were conducted across various regions: four in Canada, three in Europe (including England, Norway, and the Netherlands), two in New Zealand, and one in the United States. Six studies were international in scope, synthesizing evidence across multiple countries. There was a mix of study designs: six literature reviews, three randomized controlled trials, three quasi-experimental designs, two cross-sectional studies, and two qualitative studies. Nearly half of the studies (n = 7) focused on older adults with specific health conditions, such as diabetes and mental illness. Four studies specifically targeted older adults at a more advanced age, such as over 80. Five studies did not specify the condition of the older participants. The interventions varied in duration, ranging from a 30–40-min conversation to a three-year mail-based contact. However, four studies did not report details on the frequency or duration of the interventions.

**Table 2.** Characteristics of Included Studies Categorized by Intervention Type

Author, year of publication	Location	Aims	Study design	Study participants	Key components of the intervention	Frequency and duration	Key findings
Early identification (n = 6)							
Bannenberg et al. (2021)	Norway	Evaluate the impact of preventive home visits for older adults	Difference-in-difference	Age 80+; n = 4,056	Information and referral	60-80 min, with repeated visits offered as needed	Decreased nursing home utilization; non-significant increase in home care use
Bleijenberg et al. (2016)	Netherlands	Determine the effectiveness of a proactive primary care program on preserving daily functioning	Randomized controlled trial	Age 60+ (mean = 74.2); n = 3,092	Screening, geriatric assessment, care planning, and care coordination	1 year	Small sample size limited analysis of admissions to nursing homes and residential care
Kerse et al. (2014)	New Zealand	Assess whether case finding reduces disability among older patients	Randomized controlled trial	Age 75+ (mean = 80.3); n = 3,893	Screening and referral	Every year for 3 years	Increased residential care use; non-significant effects on the use of home help and personal care, with control group receiving more intensive services
King et al. (2018)	New Zealand	Evaluate the effect of a systematic case finding on healthcare use	Controlled before-after design	Age 75+ (mean = 81.3); n = 1,400	Screening, comprehensive assessment, and care coordination	2 weeks	Non-significant difference in residential care admissions between groups
Morris et al. (2019)	United States	Describe a technology-supported meal-delivery service	Descriptive analysis	Age 65+ (mean = 77.4); n = 867	Screening and referral	12 months	Of 239 alerts, drivers identified 12% for personal care; coordinators made 132 referrals, 24% for personal care
Whate et al. (2021)	Canada	Examine the performance of the interRAI ED screener in predicting adverse outcomes	Retrospective study	People aged 65+ (mean = 71.7) with less severe conditions; n = 2,810	Screening and referral	Not specified	The screener effectively predicted increased home care use in high-risk individuals
Integrated care (n = 5)							
Briggs et al. (2022)	Global	Determine the effectiveness of comprehensive geriatric assessment	Cochrane review	21 studies included; people (65+) with frailty or at risk for poor outcomes	Multidimensional interdisciplinary assessment and coordinated care plan	6-36 months, with a median follow-up of 12 months	Non-significant effect on nursing home admissions over 12 months
Deschodt et al. (2020)	Global	Identify core components of nurse-led integrated care and evaluate its effectiveness	Systematic review and meta-analysis	19 studies included, mean age = 71.8-85	Screening, holistic assessment, individualized care plan, care coordination, and referral	3-24 months	Non-significant effect on nursing home admissions
Elston et al. (2019)	England	Assess the impact of holistic link-workers for clients	Before-and-after design	People aged 50+ (mean = 79.6) with complex health needs	Information, practical support, and advocacy to access services	A 30-40-min conversation, with a 12-week follow-up	Increase in the utilization of community and social care
Weeks et al. (2018)	Global	Synthesize evidence for the impact of transitional care on healthcare utilization	Systematic review	23 studies included; people aged 60+ (mean = 76.3) with at least one medical diagnosis	Information, assessment, coordination, and support with accessing services	3-20 (mean = 7.5) contacts. Length of time: 5.8 days - 3 years (mean = 5.5 months)	Non-significant effect on nursing home admissions
Yous et al. (2023)	Canada	Explore the experiences of older adults in a self-management intervention	Qualitative (interview) study	People aged 65+ (mean = 71.7) with diabetes and at least one other chronic conditions; n = 45	Care coordination and navigation support	6 months	Older persons felt connected to community resources and were referred to social support services
Partnership (n = 2)							
Groenvynck et al. (2021)	Global	Develop a model to optimize transition from home to nursing homes	Literature review and expert consultation	20 studies included	Support, information, communication, and time	Not specified	Partnership is essential through the transition process
Ploeg et al. (2017)	Canada	Understand how family physicians facilitate access to community services for older persons	Qualitative (interview) study	23 family physicians	Information, referral, and encouragement	Not specified	Strategies to facilitate service access: Team communication, encouraging access, and initiating service linkages
**Other types (n = 3)**							
Liimatta et al. (2016)	Global	Assess the effectiveness of preventive home visits	Systematic review	19 studies included; people aged 65+	Multidimensional assessment	3-36 months	Reduced nursing home admissions
Markle-Reid et al. (2013)	Canada	Evaluate the effectiveness of nurse-led home visitation program	Randomized controlled trial	People aged 65+ (mean = 81.9) with chronic health conditions; n = 498	Multidimensional assessment, referral, and coordination	6 or 12 months	Non-significant effect on long-term care utilization
Newbould et al. (2022)	Global	Identify challenges and solutions to facilitate older adults with mental health needs to access social care	Scoping review	26 studies included; people aged 65+ with social care and mental health needs	Information and professional support	Not specified	Of the 26 included studies, only five described specific interventions to facilitate service access

Among the 16 included studies, seven explored the utilization of nursing home care (Bannenberg et al., 2021; Bleijenberg et al., 2016; Briggs et al., 2022; Deschodt et al., 2020; Groenvynck et al., 2021; Liimatta et al., 2016; Weeks et al., 2018), while six examined the use of home- and community-based services (Bannenberg et al., 2021; Elston et al., 2019; Kerse et al., 2014; Liimatta et al., 2016; Ploeg et al., 2017; Whate et al., 2021). Four studies addressed long-term care utilization without specifying service types (Markle-Reid et al., 2013; Morris et al., 2019; Newbould et al., 2022; Yous et al., 2023). Three studies investigated residential care admissions (Bleijenberg et al., 2016; Kerse et al., 2014; King et al., 2018). Additionally, almost all included studies had a broad scope beyond access to long-term care, reporting on various health outcomes and well-being indicators, such as mortality, hospital admissions, and quality of life. As this study specifically focused on interventions that facilitated access to long-term care, only the key findings related to access to such services are presented in Table 2.

The following findings are presented in two sections: intervention types and components, and key outcomes of the interventions.

### Intervention Types and Components

Based on the aims, components, and mechanisms of interventions in the included studies, we categorized them into four types (see Table 2): early identification (n = 6), integrated care (n = 5), partnership (n = 2), and other types (n = 3).

#### Early Identification

Six studies examined how early identification strategies affected access to long-term care (Bannenberg et al., 2021; Bleijenberg et al., 2016; Kerse et al., 2014; King et al., 2018; Morris et al., 2019; Whate et al., 2021). These interventions were conducted at the population level, targeting entire older populations within specific geographical areas—such as primary care practices—rather than focusing on individuals or small groups. Most of these interventions were delivered by healthcare professionals, including nurses and general practitioners (GP), while one study by Morris et al. (2019) leveraged meal delivery drivers to identify older adults at high risk and employed care coordinators without specialized training to facilitate access to care services. The primary aims of these interventions were to identify high-risk older adults at an early stage, ensure access to appropriate services, and prevent the escalation of care needs and associated high-cost healthcare utilization.

Table 3 summarizes 10 key components of the interventions identified across different intervention types. Interventions categorized as early identification mainly employed six strategies: (1) screening high-risk individuals, (2) providing service information, (3) referring individuals to appropriate services, (4) assessing individuals’ holistic needs, (5) creating personalized care plans, and (6) coordinating care services.

**Table 3.** Key Components of the Interventions by Each Service Type

Dimension	Components	Description	Early identification	Integrated care	Partnership	Other types
Approachability	Screening	Proactive identification of individuals with high needs by designated staff	√	√		
	Information	Information about available services and application procedures, potentially tailored to individual cultural, linguistic, and personal needs	√	√	√	√
Availability & accommodation	Referral	Referring individuals with care needs to appropriate services by designated staff	√	√	√	√
	Practical support	Tangible assistance in overcoming barriers to service access, e.g. advice, advocacy, system navigation, and ongoing follow-up		√	√	√
	Time	Adequate time to ensure continuity of care and enhance preparedness for receiving care			√	
Appropriateness	Assessment	Multidisciplinary evaluation of an individual’s holistic needs, including medical conditions, physical and cognitive capacities, psychological well-being, and environmental factors	√	√		√
	Care planning	Creating a personalized care plan to meet the individuals’ needs and preferences	√	√		
	Care coordination	Collaboration among services and providers to provide comprehensive care	√	√	√	√
Acceptability	Emotional support	Providing encouragement and understanding to build confidence for individuals/families accessing care, e.g. compassion from healthcare professionals			√	
	Communication	Open, comprehensive, and timely dialogue among all stakeholders, addressing key matters for older adults and families, e.g. concerns, service preferences, and peer experience sharing			√	

#### Integrated Care

Five studies assessed how integrated care facilitated access to long-term care (Briggs et al., 2022; Deschodt et al., 2020; Elston et al., 2019; Weeks et al., 2018; Yous et al., 2023). Three of them used systematic reviews or meta-analyses (Briggs et al., 2022; Deschodt et al., 2020; Weeks et al., 2018), while Elston et al. (2019) employed a quasi-experimental design, and Yous et al. (2023) adopted a qualitative interview approach. In four studies, interventions were implemented by interdisciplinary or inter-professional teams, such as nurses, GPs, therapists, and social workers, to address the holistic needs of older adults (Briggs et al., 2022; Deschodt et al., 2020; Weeks et al., 2018; Yous et al., 2023). By contrast, Elston et al. (2019) used care coordinators who were not healthcare professionals but received relevant training to support service delivery. These interventions aimed to coordinate health and social care, address complex needs of older adults, and reduce unnecessary, expensive healthcare utilization. These studies encompassed a wide range of coordinated services, spanning from primary, secondary, and acute care to long-term care and community services such as occupational therapy and physiotherapy.

Integrated care interventions typically used seven strategies (Table 3). These included: (1) screening, (2) information provision, (3) care referrals, (4) practical support, (5) comprehensive needs assessment, (6) care planning, and (7) care coordination.

#### Partnership

Two studies highlighted the importance of partnership models in facilitating access to long-term care (Groenvynck et al., 2021; Ploeg et al., 2017). Partnership refers to shared decision-making and collaborative working relationships among different stakeholders. Unlike the studies on integrated care services, which primarily evaluated the effectiveness of interventions, these two studies focused on identifying and reporting the processes involved in forming successful partnerships rather than measuring intervention outcomes. Although their impacts were not directly evaluated, these two studies suggested that the interventions shared a broader goal of promoting access to long-term care services and support.

The partnership components described in the two studies differed significantly. Groenvynck et al. (2021) emphasized fostering a partnership between older adults, informal caregivers and healthcare professionals, though the specific roles of healthcare professionals were not explicitly defined. They identified four key components essential for a successful partnership throughout the transition trajectory (i.e. pre-, mid-, and post-transition of care from homes to nursing homes), including: accurate information, clear communication, ongoing support, and sufficient time. In contrast, Ploeg et al. (2017) focused on the role of interprofessional teamwork in facilitating service access for older adults. Their partnership model involved various care professionals, such as nurses, dietitians, family physicians, social workers, and mental health workers. Although Ploeg et al. (2017) did not assess the effectiveness of the intervention, they identified several strategies adopted by family physicians to promote service access. These included collecting service information from team members, encouraging families to access services on behalf of older adults, and providing referrals for those in need of additional support.

Seven key strategies were used among the partnership interventions. These included: (1) information provision, (2) care referrals, (3) practical support, (4) adequate time, (5) care coordination, (6) emotional support, and (7) tailored communications.

#### Other Types

Three studies were categorized as other types (Liimatta et al., 2016; Markle-Reid et al., 2013; Newbould et al., 2022). These interventions were mainly implemented by healthcare professionals, such as nurses, physiotherapists, and social workers. While Newbould et al. (2022) briefly mentioned healthcare professionals in delivering the interventions, they did not explicitly define the role of healthcare professionals. In a review by Liimatta et al. (2016), health visitors and trained medical students were identified as key contributors in two studies.

These interventions mainly adopted five strategies to promote access to long-term care. These included: (1) information provision, (2) care referrals, (3) practical support, (4) comprehensive needs assessment, and (5) care coordination.

### Key Outcomes of the Interventions

Next, we present the key outcomes of the interventions by service types. Concerning early identification interventions, two studies found that they enhanced access to home-based services and residential care (Bannenberg et al., 2021; Kerse et al., 2014). These interventions achieved this by identifying unmet needs at an early stage and facilitating timely referrals to long-term care. In addition to early detection, Bannenberg et al. (2021) revealed two additional mechanisms. The intervention increased older persons’ awareness of available prevention technologies and encouraged them to adopt preventive measures to maintain health. This, in turn, contributed to a reconfiguration of service utilization patterns by reducing reliance on high-intensity nursing care and increasing the use of home-based services. While the remaining four studies yielded uncertain results regarding access to long-term care (Bleijenberg et al., 2016; King et al., 2018; Morris et al., 2019; Whate et al., 2021), they emphasized the potential benefits of early identification in improving service access. King et al. (2018) observed a non-significant reduction in residential care admissions in the intervention group, which they suggested could be attributed to the enhanced use of more appropriate rehabilitation services, facilitated by early identification. Bleijenberg et al. (2016) found inconclusive evidence on the impact of a primary care program on admissions to nursing homes and residential homes due to a small sample size. Two studies did not directly assess the effect of the interventions on service access. Morris et al. (2019) found that employing meal delivery drivers for early detection helped identify high-risk older adults and facilitated timely access to appropriate services. Whate et al. (2021) found that the use of a screening tool led to increased referrals to home care services by identifying individuals at high risk.

Among the five studies categorized as integrated care, three reported no statistically significant effect of integrated care on nursing home admissions (Briggs et al., 2022; Deschodt et al., 2020; Weeks et al., 2018). This non-significant result may be attributed to the heterogeneity and complexity of integrated care models included in these systematic reviews, which varied in components, intervention duration, and outcomes measures (Deschodt et al., 2020; Weeks et al., 2018). Additionally, Briggs et al. (2022) pointed to the potential influence of contextual factors, such as differences in nursing home admission rates across countries and the availability of home-based services. However, two studies reported increased access to long-term care and support (Elston et al., 2019; Yous et al., 2023). Elston et al. (2019) found that link-workers, who acted as care coordinators to assess older adults’ needs and connect them with appropriate services, significantly increased the use of community and social care. Similarly, Yous et al. (2023) reported positive experiences with navigation support, which successfully linked individuals to community-based services.

As partnership interventions primarily documented implementation processes, outcomes data were not reported. Regarding other types of interventions, results were mixed. Liimatta et al. (2016) reported a significant reduction in nursing home admissions, which they attributed to improved health conditions among older adults. Conversely, Markle-Reid et al. (2013) found no significant impact of the nurse-led home visitation program on long-term care utilization, possibly due to an insufficient sample size and statistical power. Newbould et al. (2022) discussed strategies to address resistance to receiving long-term care among older adults with mental illnesses, particularly those with dementia. Instead of focusing solely on the behavioral symptoms of older adults living with dementia, Newbould et al. (2022) recommended a shift in intervention design to prioritize external factors, such as tailoring services to suit individual preferences and increasing public funding to provide more services.

## Discussion

This scoping review aimed at mapping the literature on interventions that promoted access to long-term care for community-dwelling older adults. From the 16 included studies, four broad categories of interventions were identified: early identification, integrated care, partnership, and other types. Across these categories, three commonly adopted components included providing information, facilitating referrals, and offering care coordination. These findings expand the literature regarding how to help older adults living in the community access timely and appropriate long-term care services and support, potentially improving their well-being and reducing costly healthcare utilization.

Our findings reveal mixed evidence on the effectiveness of the identified interventions in promoting access to long-term care. Notably, there is a lack of studies specifically examining the utilization of long-term care. Instead, most research focuses more broadly on healthcare utilization or older adults’ well-being. This broader focus may explain why nursing home usage—sometimes classified as a healthcare service due to its provision of medical services—was the most commonly reported outcome in the studies reviewed. Among the four intervention types identified by our review, early identification models appear particularly promising for enhancing access to long-term care. These models adopted proactive, population-level strategies to identify high-risk older adults early and facilitate timely referrals to appropriate services (Bannenberg et al., 2021; Morris et al., 2019). This approach aligns with the finding from Stephan et al. (2018), which underscored the crucial role of early contact by healthcare professionals in facilitating access to long-term care for individuals with emerging care needs.

Additionally, evidence on the impact of intervention length on the effectiveness of the intervention is limited. One key challenge is the lack of explicit reporting on both the frequency and duration of the intervention, which makes comparisons across studies difficult. Among the 16 included studies, only one examined the influence of intervention length, suggesting that brief interventions (one month or less) were as effective as longer interventions (ranging from several months to three years) in reducing costly healthcare utilization (Weeks et al., 2018). However, they did not explore whether intervention length affected access to long-term care. Overall, the relationship between the length of interventions and their effectiveness in improving service access remains unclear and warrants further research.

This review indicates that healthcare professionals, particularly nurses and GPs, play a major role in delivering interventions aimed at improving access to long-term care. Two factors may explain their substantial involvement. First, as front-line healthcare workers, nurses and GPs often serve as the initial or sole point of contact for older adults seeking information about available services (Deschodt et al., 2020; Ploeg et al., 2017). Their established rapport with older adults may increase the likelihood of participation in intervention studies. Second, using the existing workforce of healthcare professionals is a practical and cost-effective approach, especially in healthcare systems where nurses and GPs are already integral to care delivery. Nonetheless, this review also identifies the importance of involving non-healthcare professionals in facilitating access to long-term care, such as meal delivery drivers trained to detect high-risk older adults and refer them to appropriate services (Morris et al., 2019). Since these roles require less specialized expertise, targeted training could be both viable and cost-effective, especially in settings facing healthcare workforce shortages or limited resources.

While existing interventions aimed at facilitating long-term care service access for older adults varied in their targeted populations, two-thirds of included studies explicitly focused on older adults with advanced age or specific health conditions (Bannenberg et al., 2021; Briggs et al., 2022). This highlights the importance of tailoring interventions to those more likely to need support. However, due to the heterogeneity in interventions components and participant characteristics, it remains unclear whether older adults at high risk are more likely to benefit than the general older population. (Duan-Porter et al., 2020) suggested that while interventions targeting individuals with fewer impairments may be beneficial, they often require long-term, large-scale investments at the population level to achieve significant benefits. Therefore, in resource-constrained settings, future research and practice could prioritize designing interventions specifically targeting older adults at high risk or with complex health needs, such as multimorbidity and frailty, to maximize the impact of available resources in enhancing access to long-term care.

We identified four types of interventions that promoted access to long-term care, with early identification emerging as a potentially effective approach. Given the main purpose of scoping reviews is to map broader, descriptive aspects of the literature rather than evaluating the effectiveness of interventions (Peters et al., 2022), we are cautious about drawing conclusions on their effectiveness and highlight the need for future research. Furthermore, this scoping review identified 10 key components across service models, which we categorized into four dimensions. Each intervention included multiple components, making it challenging to disentangle which specific component is more effective or to pinpoint the underlying mechanisms that facilitate service access. This complexity may also reflect that single-component interventions are less effective in achieving desired outcomes. Additionally, our findings indicate that offering information, referrals, and coordinated care are widely used across the four types of interventions. Future research could examine how these commonly used components operate, who benefits most from them, and the specific contexts under which they are most effective.

### Limitations

This scoping review has some limitations. First, only articles published in English were included, which might exclude relevant studies in other languages and narrow the perspective on this topic. Second, this review focused on peer-reviewed articles, omitting grey literature. A future review of the grey literature in this area could provide additional insights into strategies for promoting access to long-term care. Third, due to the large volume of hits generated by broad terms (e.g., service, program, and policy), we excluded them from our search strategy. This may overlook some interventions. Future work could explore refined search approaches for such terms. Lastly, a formal quality assessment of the included studies was not conducted because the primary aim of this scoping review was to provide an overview of existing interventions rather than evaluate methodological rigor (Peters et al., 2022).

## Conclusions and Implications

This scoping review highlighted the need for designing and evaluating interventions that can promote access to long-term care services and support for community-dwelling older adults. While four types of interventions were identified, most interventions were developed specifically for maintaining health conditions and reducing costly healthcare utilization, rather than explicitly enhancing access to long-term care. The interventions varied widely in their components and duration, which prevented us from recommending or discouraging specific approaches to facilitate service access. However, common characteristics emerged across the interventions. Many highlighted outcomes related to nursing home admissions rather than the use of other long-term care services. Additionally, most older adults were at high risk and relied on healthcare professionals for implementation. Importantly, we acknowledge the feasibility and potential benefit of leveraging non-healthcare professionals to deliver cost-effective interventions, particularly in resource-constrained settings. Future research could investigate the effectiveness of interventions delivered by non-healthcare professionals in improving service access, and identify which older adults with varying individual characteristics are more likely to benefit from these interventions. To the best of our knowledge, this is the first scoping review to examine interventions that facilitate access to long-term care for community-dwelling older adults.

## Supplemental Material

Supplemental material - Interventions to Facilitate Access to Long-Term Care for Community-Dwelling Older Adults: A Scoping Review Supplemental material for Interventions to Facilitate Access to Long-Term Care for Community-Dwelling Older Adults: A Scoping Review by Jinbao Zhang, Alan Dargan, Wenjing Zhang, Julien Forder in Research on Aging.

## Data Availability

Data used for synthesis is available from the included studies. This review was pre-registered on OSF (registration link: https://doi.org/10.17605/OSF.IO/PQ4UT) (Zhang et al., 2023).
